# Cord Blood 25(OH)-Vitamin D Deficiency and Childhood Asthma, Allergy and Eczema: The COPSAC_2000_ Birth Cohort Study

**DOI:** 10.1371/journal.pone.0099856

**Published:** 2014-06-12

**Authors:** Bo L. Chawes, Klaus Bønnelykke, Pia F. Jensen, Ann-Marie M. Schoos, Lene Heickendorff, Hans Bisgaard

**Affiliations:** 1 Copenhagen Prospective Studies on Asthma in Childhood, Health Sciences, University of Copenhagen, Danish Pediatric Asthma Center, Copenhagen University Hospital, Gentofte, Denmark; 2 Department of Clinical Biochemistry, Aarhus University Hospital, Aarhus, Denmark; National Taiwan University, Taiwan

## Abstract

**Background:**

Epidemiological studies have suggested an association between maternal vitamin D dietary intake during pregnancy and risk of asthma and allergy in the offspring. However, prospective clinical studies on vitamin D measured in cord blood and development of clinical end-points are sparse.

**Objective:**

To investigate the interdependence of cord blood 25-hydroxyvitamin D (25(OH)-Vitamin D) level and investigator-diagnosed asthma- and allergy-related conditions during preschool-age.

**Methods:**

Cord blood 25(OH)-Vitamin D level was measured in 257 children from the Copenhagen Prospective Studies on Asthma in Childhood (COPSAC_2000_) at-risk mother-child cohort. Troublesome lung symptoms (TROLS), asthma, respiratory infections, allergic rhinitis, and eczema, at age 0–7 yrs were diagnosed exclusively by the COPSAC pediatricians strictly adhering to predefined algorithms. Objective assessments of lung function and sensitization were performed repeatedly from birth.

**Results:**

After adjusting for season of birth, deficient cord blood 25(OH)-Vitamin D level (<50 nmol/L) was associated with a 2.7-fold increased risk of recurrent TROLS (HR = 2.65; 95% CI = 1.02–6.86), but showed no association with respiratory infections or asthma. We saw no association between cord blood 25(OH)-Vitamin D level and lung function, sensitization, rhinitis or eczema. The effects were unaffected from adjusting for multiple lifestyle factors.

**Conclusion:**

Cord blood 25(OH)-Vitamin D deficiency associated with increased risk of recurrent TROLS till age 7 years. Randomized controlled trials of vitamin D supplementation during pregnancy are needed to prove causality.

## Introduction

Vitamin D deficiency caused by adaption of a more sedentary indoor lifestyle and changing dietary habits has become a common health problem in developed and developing countries worldwide[1] which has occurred in parallel with the “asthma epidemic”[2]. It is now evident that vitamin D possesses a panoply of immune-regulatory functions that may protect against asthma and allergy [3] and recent findings also suggest a role of vitamin D for fetal lung cell maturation and subsequent lung function development [4]. These findings lend support to the hypothesis that fetal vitamin D deficiency may promote a trajectory to develop asthma and allergy [5]. A growing amount of studies have attempted to prove this hypothesis but with inconsistent findings.

Some epidemiological studies have shown an association between low maternal vitamin D intake during pregnancy and increased risk of wheezy phenotypes in the offspring [6], [7] whereas others showed association with lower respiratory tract infections but not with wheezy symptoms [8] or asthma [8], [9]. Likewise, some studies have shown that fetal vitamin D deficiency increases the risk of food allergy [10] and eczema [11], [12] whereas others found no association with allergic sensitization [12]. Thus, it remains uncertain whether fetal vitamin D deficiency contributes to the development of childhood asthma, allergy and eczema. This may be a result from the inaccuracy of determining fetal Vitamin D exposure by questionnaire-based estimations of maternal dietary intake of vitamin D and/or poorly defined case definitions.

The aim of the current study was to investigate the programming effect of cord blood vitamin D deficiency on the subsequent development of troublesome lung symptoms (TROLS), asthma, lower respiratory tract infections, lung function, allergy, and eczema during preschool age. We studied these aspects in the children from the Copenhagen Prospective Study on Asthma in Childhood (COPSAC_2000_) high-risk birth cohort [13]–[15] with longitudinal clinical data determined from strict predefined algorithm-based clinical case definitions and repeated objective assessments of intermediary end-points.

## Materials and Methods

### Study Design

The COPSAC_2000_ birth cohort is a single-center prospective clinical study of 411 children born to mothers with physician verified asthma recruited between 1998 and 2001 as previously described [13]–[15]. The children were enrolled at one month of age and subsequently attended the clinical research unit for scheduled clinical investigations at six-monthly intervals as well as immediately upon onset of any respiratory-, allergy- or skin-related symptom. The pediatricians employed at the clinical research unit (not the family practitioners) were solely responsible for diagnosis and treatment of asthma, allergy, and eczema. At every visit a full physical examination was performed including lung function testing and history was obtained by parental interviews using predefined questions with closed response categories. History information was collected online during the visits and the objective measurements were double checked against source data and subsequently locked.

The study was conducted in accordance with the Declaration of Helsinki and was approved by The Copenhagen Ethics Committee (KF 01-289/96) and The Danish Data Protection Agency (2008-41-1754). Written informed consent was obtained from both parents before enrollment.

### Cord Blood Vitamin D Measurement

Cord blood was collected by the midwives by needle puncture from the umbilical cord vein obtaining an aliquot of approximately 14 mL which was subsequently sent to the COPSAC research unit, centrifuged for 10 min at 4300 rpm to separate serum, and thereafter frozen at −80°C until analysis.

The serum samples were transported on dry ice for duplicate analyses for 25-hydroxyvitamin D2 (25(OH)-Vitamin D2) and 25(OH)-Vitamin D3 at the Dept. of Clinical Biochemistry, Aarhus University Hospital, Denmark. Serum 25-hydroxyvitamin D levels were analyzed by isotope dilution liquid chromatography-tandem mass spectrometry (LC-MS/MS) [16], [17]. Calibrators traceable to NIST SRM 972 (Chromsystems, DE) were used. Mean coefficients of variation (CV) for 25(OH)-Vitamin D3 were 6.4% and 9.1% at levels of 66.5 and 21.1 nmol/L and for 25(OH)-Vitamin D2 the CV values were 8.8% and 9.4% at levels of 41.2 and 25.3 nmol/L. The average of the combined 25(OH)-Vitamin D values was calculated and used in the analysis. If both 25(OH)-Vitamin D2 and 25(OH)-Vitamin D3 were under the detection level, the combined value was defined as equal to 10 nmol/L.

### Clinical Investigator-diagnosed End-points

**Troublesome lung symptoms (TROLS)** were defined as significant cough or wheeze or dyspnea and were explained to the parents as wheeze or whistling sounds, breathlessness, or recurrent troublesome cough severely affecting the well-being of the child and were recorded by the parents in a day-to-day diary chart as a dichotomized daily score (yes/no) from birth till age 7 yrs [18]. **Recurrent TROLS** was defined from the diaries at the scheduled or acute visits to the research clinic as five episodes within 6 months, each episode lasting at least three consecutive days, or daily symptoms for four consecutive weeks [19], [20]. Children meeting these criteria were prescribed a 3-month trial of budesonide 200 mcg bid increasing to 6 and 12 months at subsequent relapses.

**Asthma** at age 7 yrs was diagnosed according to international guidelines and was based on recurrent TROLS as defined above, symptoms judged by the COPSAC pediatricians to be typical of asthma (e.g. exercise induced symptoms, prolonged nocturnal cough, recurrent cough outside common cold, symptoms causing wakening at night); in need of intermittent rescue use of inhaled β_2_-agonist; responding to a 3-month trial of inhaled corticosteroids and relapsing when stopping treatment [14], [15].

**Lower respiratory tract infections (LRTI)** included occurrence of pneumonia and/or acute bronchiolitis at age 0–3 yrs where such disorders are most prevalent. The diagnoses were established at the acute visits to the clinic by the research pediatricians based on clinical appearance regardless of identified pathogen(s) in accordance with predefined standard procedures [21] or if the child had been hospitalized for such disorders.

**Allergic rhinitis** was diagnosed at age 7 yrs based on clinical interviews of the parents on history of symptoms in the child's 7^th^ year of life [22]–[24]. Rhinitis was defined as troublesome sneezing or blocked or runny nose in the past 12 months in periods without accompanying cold or flu [25].

**Eczema** was diagnosed utilizing the Hanifin-Rajka criteria as previously detailed [26], [27] obtaining age at onset data. Skin lesions were described at both scheduled and acute visits according to pre-defined morphology and localization.

### Lung Function

**Infant spirometry** was performed during sedation at age 1 month by applying the raised volume rapid thoraco-abdominal compression technique as previously detailed [28]–[30]. The forced expiratory volume at 0.5 seconds (FEV_0.5_) and forced expiratory flow at 50% of the forced vital capacity (FEF_50_) were used as lung function indices.

**Spirometry at age 7 yrs** was performed as previously detailed [31] using a pneumotachograph Masterscope Pneumoscreen, system 754,916 spirometer (Erich Jaeger, Wurtzburg, Germany) for assessing FEV_1_ and maximal mid-expiratory flow (MMEF).

**Bronchial responsiveness** at age 1 month was assessed as previously detailed [29] by continuous measurements of transcutaneuos oxygen saturation (PtcO_2_) during quadrupling methacholine dose-steps and was defined as the provocative dose causing a 15% drop in PtcO_2_ (PD_15_). Bronchial responsiveness at age 7 yrs was defined as the provocative dose of methacholine causing a 20% drop in FEV_1_ from baseline (PD_20_) [31].

### Allergy Intermediary End-points

**Specific-IgE** was measured at age ½, 1½, 4, and 6 yrs against 16 common inhalant and food allergens (cat, dog, horse, birch, timothy grass, mugwort, house dust mites, moulds, hen's egg, cow's milk, fish, wheat, peanut, soybean, or shrimp) by ImmunoCAP assay (Pharmacia Diagnostics AB, Uppsala, Sweden). Allergic sensitization was defined as specific-IgE ≥0.35 kU/L [32], [33] for (1) any of the tested allergens, (2) any inhaled allergen, and (3) any food allergen.

**Total-IgE** was measured at age ½, 1½, 4, and 6 yrs by ImmunoCAP (Pharmacia Diagnostics AB, Uppsala, Sweden) with a detection limit of 2 kU/L [33].

### Covariates

**Blood sampling season** was categorized as: winter (Dec-Feb), spring (March-May), summer (June-Aug) and fall (Sep-Nov). *Demographics* included child gender, birth BMI, maternal age at birth of proband, and household income (low: <50,000€, medium: 50,000–80,000€, high>80,000€). **Postnatal exposures** were older siblings (yes/no), length of solely breastfeeding, age at start in daycare, and environmental tobacco exposure measured objectively as hair nicotine level at age 1 yr [34].

### Statistical Analysis

The combined cord blood 25(OH)-Vitamin D value was analyzed as a continuous variable per 100 nmol/L decrease as well as categorized as: deficient (<50 nmol/L), insufficient (50–75 nmol/L), sufficient (>75 nmol/L), based on biologically relevant levels according to recent studies on multiple health outcomes [1].

Distribution of baseline characteristics within the study group and the drop-out analysis was done with univariable parametric and non-parametric tests such as χ^2^, Fischer's exact test, t-test, and Kruskal-Wallis rank sum test.

The association of cord blood 25(OH)-Vitamin D level with age at onset end-points (recurrent TROLS, LRTI, eczema) was estimated as hazard ratios by Cox regression and visualized by Kaplan-Meier curves for the categorized 25(OH)-Vitamin D levels. The effect of 25(OH)-Vitamin D level on the cross-sectional end-points, e.g. asthma and allergic rhinitis, was estimated as odds ratios by logistic regression. The association between 25(OH)-Vitamin D and cross-sectional continuous outcomes such as lung function incentives was explored by general linear models (GLM). The effect on outcome measures with repetitive assessments (allergic sensitization, total-IgE) was modeled using general estimating equations (GEE) applied to compute the overall odds ratio utilizing compiled data from all four time points (½, 1½, 4, 6 yrs). PD_15_, PD_20_ and total-IgE were log-transformed and z-scores were calculated for FEV_0.5_, FEV_1_, FEF_50_ and MMEF prior to analyses.

Additional adjusted effect estimates were calculated with multivariable models including all covariates significantly associated with cord blood 25(OH)-Vitamin D level (p≤0.05) in the models. All analyses were adjusted for blood sampling season [12]. Results are reported with 95% confidence intervals (CI) in brackets, a p-value≤0.05 is considered significant. Analyses were done using SAS version 9.2 (SAS Institute, Cary, NC).

The core data of the manuscript is available online in **Data S1**.

## Results

### Baseline Characteristics

Cord blood was available for 25(OH)-Vitamin D analysis in 257 (63%) of the 411 children in the COPSAC_2000_ cohort. Characteristics of the children with and without available cord blood samples are given in Table 1. Children in the drop-out group without available cord blood came from families with significantly lower household income (p = 0.01), but did not differ from the study group in any other baseline characteristics studied.

**Table 1:** Baseline characteristics of children with and without available cord blood for 25(OH)-Vitamin D analysis.

	Cord blood 25(OH)-Vitamin D (nmol/l), N = 257				No cord blood available, N = 154	
	Deficient: <50	Insufficient: 50–75	Sufficient:>75	P*		P**
**Vitamin D cord blood** (N)	53% (136)	32% (82)	15% (39)	-	-	-
**DEMOGRAFICS**						
Maternal age at birth^1^, median (range)	29.5 yrs (20.9–38.9)	29.2 yrs (22.6–41.1)	30.8 yrs (22.1–37.8)	**0.02**	30.0 (19.2–41.2)	0.31
Household income^2^ (N)				0.25		**0.01**
Low, <50.000E	28% (35)	25% (19)	13% (5)		37% (53)	
Medium, 50.000–80.000E	52% (65)	55% (42)	53% (20)		38% (55)	
High, >80.000E	21% (26)	20% (15)	34% (13)		25% (36)	
Boy^2^ (N)	45% (61)	55% (45)	46% (18)	0.34	51% (79)	0.55
Birth BMI1, median (range)	12.8 kg/m^2^ (7.0–16.3)	12.7 kg/m^2^ (10.0–16.6)	12.8 kg/m^2^ (9.6–15.1)	0.98	12.6 kg/m^2^ (8.4–16.5)	0.35
Birth season^1^ (N)				**<0.01**		0.39
Winter	30% (41)	15% (12)	18% (7)		23% (35)	
Spring	24% (33)	21% (17)	15% (6)		19% (30)	
Summer	21% (28)	33% (27)	49% (19)		24% (37)	
Fall	25% (34)	32% (26)	18% (7)		34% (52)	
**POSTNATAL EXPOSURES**						
Older siblings^2^ (N)	44% (55)	26% (20)	50% (19)	**0.02**	40% (58)	0.87
Breastfeeding^3+4^, median (range)	123 (0–243)	121 (0–274)	137 (0–266)	0.14	120 (0–244)	0.14
Daycare^4+5^, median (range)	339 (140–1074)	308 (156–674)	286 (179–578)	0.46	355 (127–1003)	0.16
Nicotine in hair 1 yr^4^, median (range)	0.84 ng/mg (0.06–30.9)	0.53 ng/mg (0.03–43.5)	0.47 ng/mg (0.1–12.9)	**0.03**	0.95 (0.03–103.9)	0.14

*P-value for the distribution of characteristics within the groups of 25(OH)-Vitamin D.

**P-value for the distribution of characteristics between children with and without available 25(OH)-Vitamin D data.

1 Linear regression; ^2^Chi-Square test; ^3^Days solely breastfed; ^4^Kruskal-Wallis rank sum test; ^5^Age at start in daycare.

The median cord blood 25(OH)-Vitamin D level in the study group was 47.6 nmol/L (range, 10–145 nmol/L). The distribution of cord blood 25(OH)-Vitamin D concentrations was: 132 (53%) children with deficient levels, 82 (32%) with insufficient, and 39 (15%) with sufficient levels. Concentration of cord blood 25(OH)-Vitamin D varied significantly with season of birth (p<0.01) with most 25(OH)-Vitamin D deficient children born during winter and most sufficient children born during summer. Children with deficient vs. sufficient levels were born to younger mothers (median age at birth, 29.5 yrs vs. 30.8 yrs; p = 0.02), had fewer siblings (children with older siblings, 44% vs. 50%; p = 0.02), and had a higher exposure to environmental tobacco smoke (median hair nicotine level at age 1 yr, 0.84 vs. 0.47 ng/mg; p = 0.03).

### Cord Blood 25(OH)-Vitamin D Level vs. Asthma-related Outcomes and LRTI

**Figure 1:**
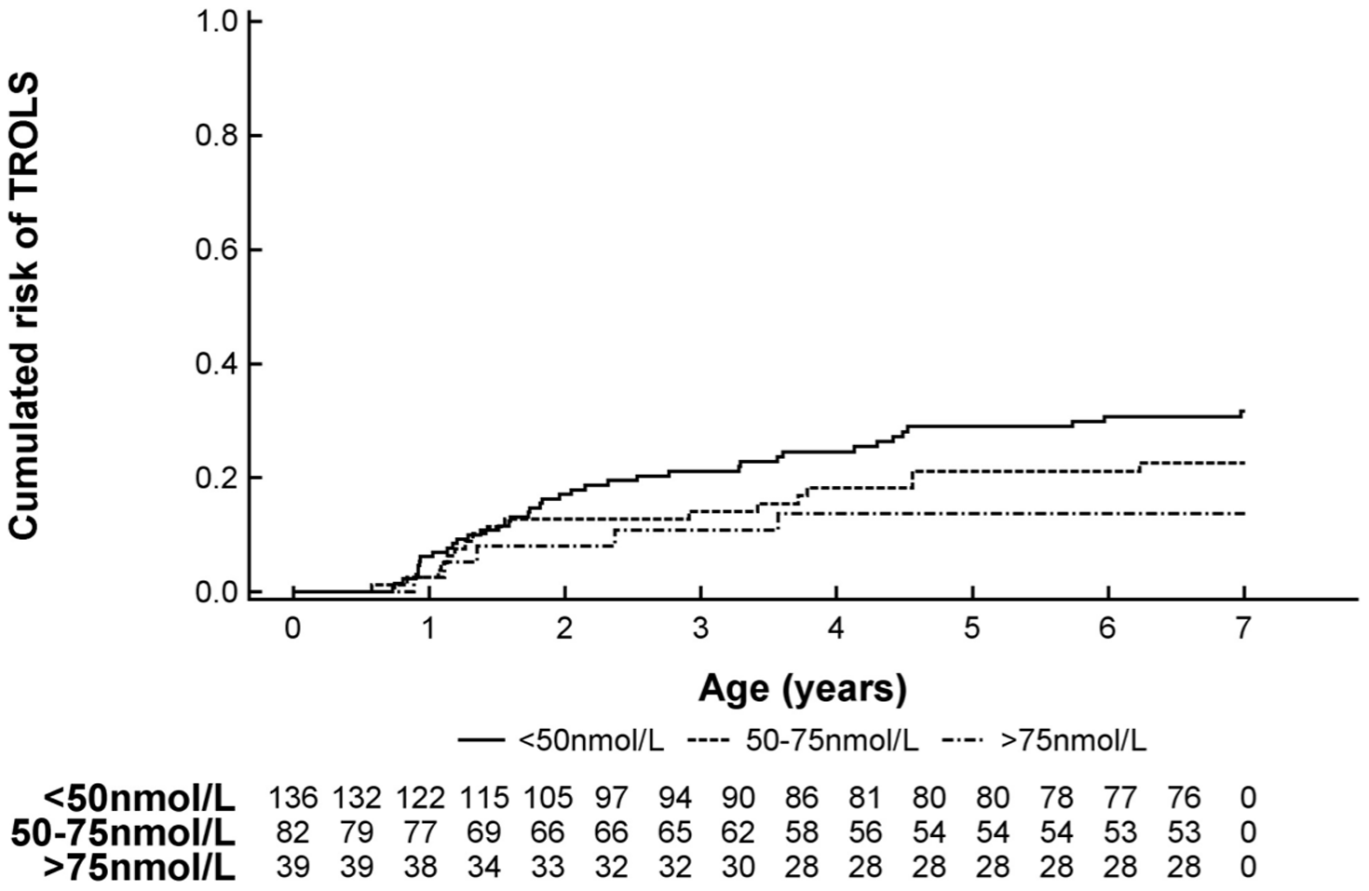
Kaplan Meier survival curve showing the risk of developing recurrent troublesome lung symptoms (TROLS) at age 0–7 yrs stratified by cord blood 25(OH)-Vitamin D level.

Recurrent TROLS was diagnosed in 24% (N = 61) of the children at age 0–7 yrs. The cumulative prevalence rates were 4% (N = 11) at age 0–1 yrs, 14% (N = 35) at 0–2 yrs, 16% (N = 42) at 0–3 yrs, 19% (N = 50) at 0–4 yrs, 22% (N = 57) at 0–5 yrs, and 23% (N = 59) at 0–6 yrs. Cord blood 25(OH)-Vitamin D levels were lower in children developing recurrent TROLS as illustrated in Figure 1. Having deficient vs. sufficient cord blood 25(OH)-Vitamin D level was associated with a 2.7-fold increased risk of recurrent TROLS (hazard ratio (HR), 2.65; 95% CI = 1.02–6.86; **p = 0.04**). The association was borderline significant when adjusting for multiple confounding factors but with unchanged effect estimates (aHR, 2.50; 95% CI, 0.95–6.57; p = 0.06).

**Asthma** at age 7 yrs was diagnosed in 16% (N = 34) of the children. 25(OH)-Vitamin D levels in cord blood showed no association with asthma at age 7 yrs (**Table 2**). In addition, cord blood 25(OH)-Vitamin D level was not suppressed in the subgroup of children experiencing recurrent TROLS who were subsequently diagnosed with asthma at age 7 yrs (N = 31): OR 2.25 (95% CI, 0.60–8.49; p = 0.21) for deficient vs. sufficient levels.

**Table 2:** Cord blood 25(OH)-Vitamin D vs. asthma-related outcomes, lower respiratory tract infections and lung function.

	Cord blood 25(OH)-Vitamin D							
	<50 vs >75 nmol/L	P	<50 vs >75 nmol/L Adjusted*	P	Per 100 nmol/L decrease	P	Per 100 nmol/L decrease Adjusted*	P
**Asthma-related outcomes**								
Recurrent TROLS, 0–7 yrs^1+2^	**2.65 (1.02 to 6.86)**	**0.04**	**2.50 (0.95 to 6.57)**	**0.06**	**2.65 (0.83 to 8.50)**	**0.10**	2.27 (0.65 to 7.90)	0.20
Asthma, 7 yrs^3^	1.60 (0.49 to 5.22)	0.31	1.63 (0.47 to 5.61)	0.38	1.18 (0.25 to 5.58)	0.84	1.01 (0.18 to 5.80)	0.99
**LRTI^4^**								
Time to first LRTI, 0–3 yrs	1.08 (0.63 to 1.85)	0.78	1.08 (0.62 to 1.87)	0.79	1.17 (0.54 to 2.52)	0.69	1.05 (0.46 to 2.39)	0.91
No. of LRTI, 0–3 yrs^5^	1.07 (0.66 to 1.75)	0.78	0.94 (0.57 to 1.55)	0.81	1.08 (0.53 to 2.20)	0.83	0.75 (0.35 to 1.58)	0.69
**Lung function**								
z-FEV0.5, 1mo^6+7^	0.02 (−0.10 to 0.15)	0.73	−0.01 (−0.14 to 0.13)	0.94	0.01 (−0.02 to 0.04)	0.40	0.01 (−0.02 to 0.04)	0.70
z-FEV1, 7 yrs	0.04 (−0.09 to 0.18)	0.54	0.07 (−0.08 to 0.22)	0.37	0.02 (−0.02 to 0.06)	0.28	0.02 (−0.01 to 0.06)	0.24
z-FEF50, 1mo	−0.03 (−0.15 to 0.09)	0.63	−0.06 (−0.18 to 0.07)	0.38	0.01 (−0.02 to 0.04)	0.42	0.00 (−0.03 to 0.03)	0.82
z-MMEF, 7 yrs	−0.04 (−0.17 to 0.09)	0.55	−0.05 (−0.18 to 0.09)	0.50	0.00 (−0.03 to 0.03)	0.90	−0.01 (−0.04 to 0.02)	0.62
logPD15, 1mo^8^	0.02 (−0.05 to 0.09)	0.62	0.01 (−0.07 to 0.03)	0.92	0.01 (−0.01 to 0.02)	0.66	0.00 (−0.01 to 0.02)	0.82
logPD20, 7 yrs^9^	0.02 (−0.08 to 0.12)	0.68	0.03 (−0.07 to 0.14)	0.52	0.01 (−0.02 to 0.04)	0.41	0.01 (−0.02 to 0.04)	0.40

*Adjusted for mother's age at birth, blood sampling season, older siblings, and hair nicotine level at age 1 yr.

1 TROLS = Troublesome lung symptoms; ^2^Cox regression: hazards ratio (95% CI); ^3^Logistic regression: odds ratio (95% CI); ^4^Lower respiratory tract infections; ^5^Poisson regression: incidence risk ratio (95% CI); ^6^All lung function analyses are done with general linear models: β-coefficents (95% CI); ^7^z refers to the calculated z-score; ^8^PD_15_ refers to the provocative dose of methacholine resulting in a 15% decrease in transcutaneuos oxygen saturation (PtcO_2_) from baseline; ^9^PD_20_ refers to the provocative dose of methacholine resulting in a 20% decrease in FEV_1_ from baseline.

**Figure 2:**
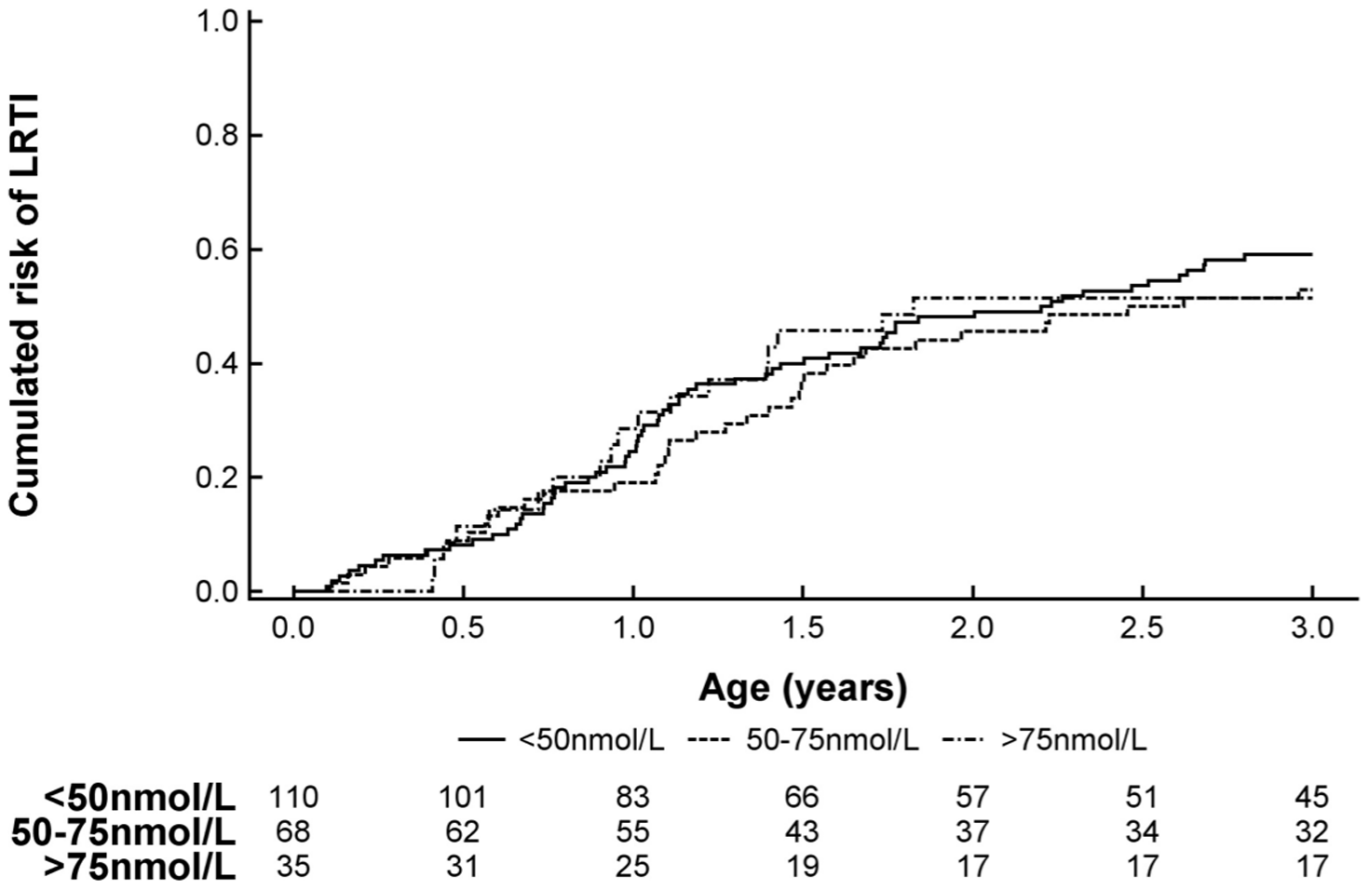
Kaplan Meier survival curve showing the risk of developing lower respiratory tract infections (LRTI) at age 0–3 yrs stratified by cord blood 25(OH)-Vitamin D level.

**LRTI** occurred in 56% (N = 119) of the study group with a mean of 1.2 (SD, 1.6) episodes per child till age 3 yrs. Cord blood 25(OH)-Vitamin D deficiency did not modify time to first LRTI (**Figure 2**) nor was the number of episodes affected by cord blood 25(OH)-Vitamin D level (**Table 2**).

### Association between Cord Blood 25(OH)-Vitamin D Level and Lung Function

Cord blood 25(OH)-Vitamin D level was not associated with forced flows assessed at age 1mo (FEV_0.5_, FEF_50_) or 7 yrs (FEV_1_, MMEF) nor bronchial responsiveness to methacholine at any age point (PD_15_, PD_20_) (Table 2).

### Relationship between Cord Blood 25(OH)-Vitamin D and Allergic Outcomes (Table 3)

#### Specific-IgE

Allergic sensitization at any of the four measuring points (½, 1½, 4, 6 yrs) was present in 35% (N = 88) for any of the investigated allergens, in 26% (N = 64) for any food allergens, and in 21% (N = 52) for any inhaled allergen. There was no significant association between cord blood 25(OH)-Vitamin D level and allergic sensitization throughout childhood although there was a tendency of Vitamin D deficiency being associated with decreased risk of sensitization.

#### Total-IgE

Cord blood 25(OH)-Vitamin D level was not associated with level of total-IgE throughout childhood.

Allergic rhinitis was diagnosed in 12% (N = 23) at age 7 yrs. Deficient vs. sufficient cord blood 25(OH)-Vitamin D level seemed to be associated with a reduced risk of allergic rhinitis at age 7 yrs but this was not significant.

### Cord Blood 25(OH)-Vitamin D Level and Risk of Eczema

**Figure 3:**
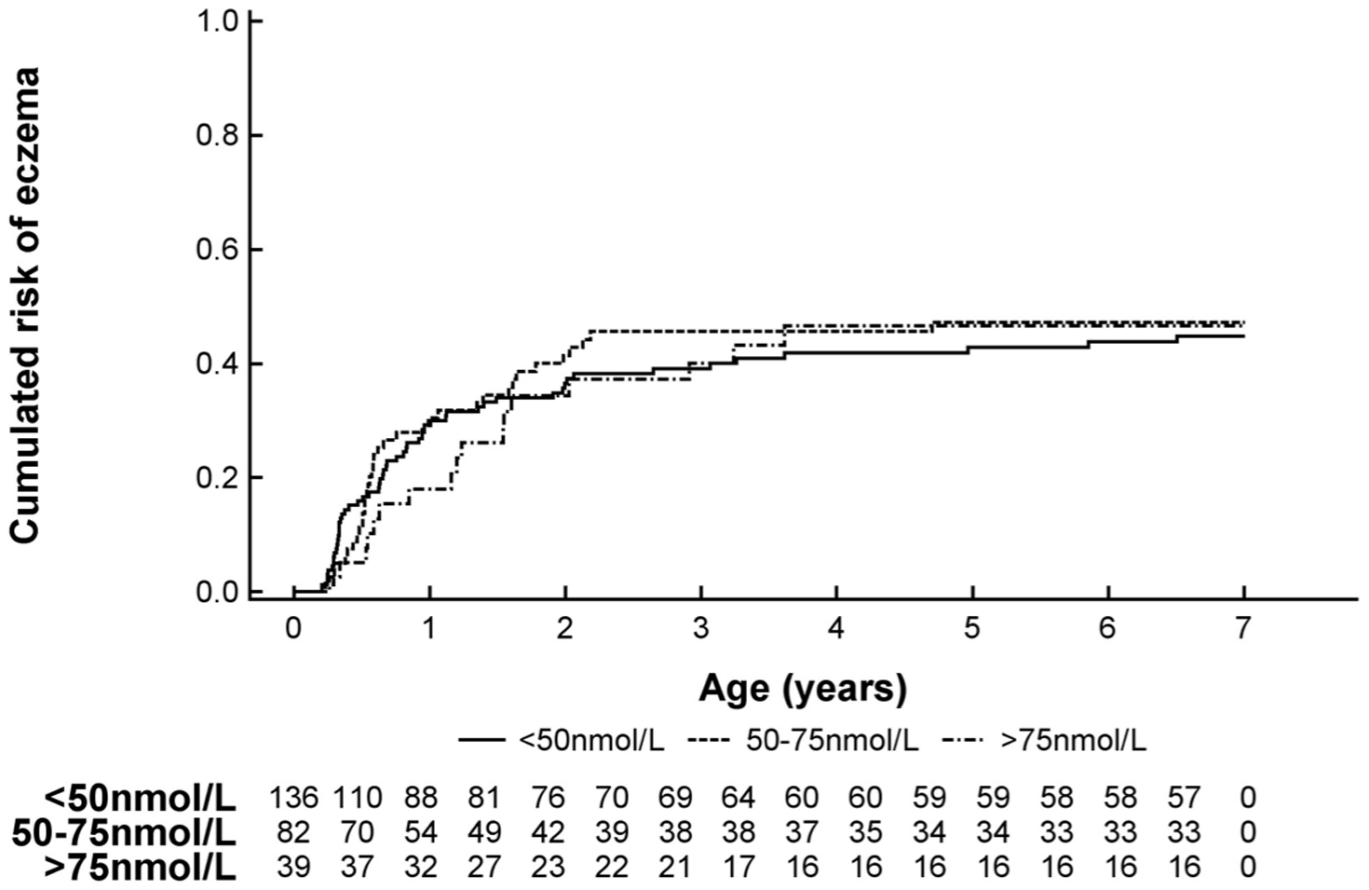
Kaplan Meier survival curve showing the risk of developing eczema at age 0–7 yrs stratified by cord blood 25(OH)-Vitamin D level.

Eczema was diagnosed in 42% (N = 109) of the study group. Cord blood 25(OH)-Vitamin D level was not significantly associated with the risk of eczema during preschool age although the effect estimates suggested an increased risk by deficient cord blood levels (See Table 3 and Figure 3).

**Table 3:** Cord blood 25(OH)-Vitamin D vs. allergic outcomes and eczema at 0–7 yrs.

	Cord blood 25(OH)-Vitamin D							
	<50 vs >75 nmol/L	P	<50 vs >75 nmol/L Adjusted*	P	Per 100 nmol/L decrease	P	Per 100 nmol/L decrease Adjusted*	P
**Allergic outcomes**								
Any sensitization, spec-IgE, 0–6 yr^1+2^	0.96 (0.43–2.16)	0.93	0.96 (0.64–2.31)	0.93	0.52 (0.17–1.52)	0.23	0.64 (0.19–2.12)	0.47
Food sensitization, spec-IgE, 0–6 yr^2^	0.86 (0.34–2.18)	0.74	0.94 (0.35–2.53)	0.91	0.47 (0.13–1.68)	0.25	0.63 (0.16–2.57)	0.53
Inhaled sensitization, spec-IgE, 0–6 yr^2^	0.97 (0.47–2.01)	0.94	1.20 (0.54–2.67)	0.65	0.36 (0.09–1.55)	0.17	0.43 (0.09–2.05)	0.29
Total-IgE, 0–6 yr^2^	0.98 (0.66–1.45)	0.91	0.98 (0.65–1.48)	0.93	0.86 (0.46–1.60)	0.63	0.83 (0.42–1.65)	0.60
Allergic rhinitis, 7 yr^3^	0.40 (0.12–1.26)	0.32	0.44 (0.13–1.49)	0.47	0.33 (0.06–1.92)	0.22	0.29 (0.04–2.14)	0.23
**Eczema**								
Time to eczema, 0–7 yr^4^	0.97 (0.56–1.69)	0.97	1.13 (0.63–2.01)	0.69	0.88 (0.41–1.92)	0.76	1.04 (0.44–2.41)	0.94
Eczema, 7 yr^3^	1.80 (0.56–5.79)	0.68	2.00 (0.59–6.76)	0.94	1.39 (0.33–5.82)	0.65	1.25 (0.27–5.82)	0.78

*Adjusted for mother's age at birth, blood sampling season, older siblings, and hair nicotine level at age 1 yr.

1 Specific-IgE≥0.35 kU/mL against any of: dog, cat, horse, birch, timothy grass, mugwort, house dust mites, molds, milk, egg, wheat, soya bean, cod and peanut; ^2^GEE model including data from age ½, 1½, 4 and 6 yrs: OR (95% CI); ^3^Logistik regression: odds ratio (95% CI); ^4^Cox regression: hazard ratio (95% CI).

## Discussion

### Principal Findings

Deficient cord blood 25(OH)-Vitamin D level was associated with a more than doubled risk of developing TROLS during preschool age in children of the Danish COPSAC_2000_ high-risk birth cohort study. This finding supports the theory that deficient 25(OH)-Vitamin D exposure in utero has a programming effect on the immune maturation thus predisposing to development of chronic inflammation.

### Strengths and Limitations of the Study

The primary strength of the study is the seven years of clinical assessments of our birth cohort with longitudinal clinical deep phenotyping and standardized diagnoses solely performed by the COPSAC research pediatricians. Repeated objective measurements along with day-to-day respiratory symptom diary recordings through the first seven years of life provided strong age at onset data which is a major advantage compared to studies using cross-sectional unspecific community-based diagnoses [6], [7], [11].

Another significant strength is the objective assessment of fetal 25(OH)-Vitamin D exposure by measuring the concentration in the cord blood. Even though cord blood 25(OH)-Vitamin D level primarily reflects recent exposure during 3^rd^ trimester and may be contaminated by maternal blood, this biological measure is a more accurate estimate of fetal 25(OH)-Vitamin D exposure compared to approximations from questionnaires on maternal dietary and supplementary intake since only 10% of vitamin D is obtained through diet [35].

It is also a strength of the analyses that environmental exposure assessments were comprehensive including hair nicotine level [34] as a validated objective measure of tobacco smoke exposure allowing for robust confounder adjustment. In agreement with other studies we found a marked seasonal variation in cord blood 25(OH)-Vitamin D level [12] as well as significant influence of various lifestyle factors such as passive smoking, maternal age at delivery, and older siblings [6], [7]. We adjusted for season of blood sampling (i.e. sunlight exposure) which is a major determinant of serum 25(OH)-Vitamin D level [12]. We did not adjust our analyses for ethnicity [1] as 98% of the study group is of Caucasian origin.

The study is limited from the at-risk nature of the cohort as all mothers have a history of asthma which may limit the generalizability of our findings to other populations. However, this should not affect our ability to analyze the relationship between 25(OH)-Vitamin D levels and development of asthma and allergy-related traits within the cohort, and our findings are in line with studies of unselected populations [7], [11].

We expected to see the largest effects in children with deficient compared to sufficient cord blood 25(OH)-Vitamin D levels. An essential limitation of the study is therefore the study sample size as only 15% had sufficient cord blood levels, equivalent to 39 participants, which results in lack of statistical power and a likelihood of overlooking the true effect of 25(OH)-Vitamin D exposure in fetal life on development of childhood asthma and allergy.

### Interpretation

Our finding that deficient cord blood 25(OH)-Vitamin D level was associated with a more than doubled risk of developing recurrent TROLS during preschool age is in line with a range of epidemiological studies utilizing maternal dietary vitamin D intake during pregnancy as a surrogate measure of fetal vitamin D exposure [6], [7], [36]. However, studies of cord blood 25(OH)-Vitamin D are still sparse [9], [11], [12] and our study is the only one with a full clinical follow-up till age 7 yrs at a single research unit employed solely with research pediatricians. Among available cord blood studies, one study found no association with recurrent wheeze [12], but only assessed the children at age 1 yr where such a diagnosis is quite infrequent in an unselected population. In line with our findings, an increased risk of deficient levels has been reported for early transient wheeze [11] and for development of wheeze till age 5 yrs [9].

It has been speculated that deficient neonatal vitamin D level mainly increases the risk of early transient wheezing exerting the effect through immune-modulatory mechanisms [3] which increase the frequency of respiratory tract infections and thus virus-induced wheezing [8], [9]. We found that low cord blood level was associated with a significantly increased risk of early recurrent TROLS, but saw no association with occurrence of early childhood respiratory infections.

We found no significant association between cord blood 25(OH)-Vitamin D level and subsequent development of allergic sensitization or allergic rhinitis although the effect estimates showed a trend of decreased risk of allergic outcomes by deficient Vitamin D levels which has also been suggested by others [37].

We did not see any significant association between fetal vitamin D exposure and development of eczema although low cord blood levels seemed to associate with an increased risk. This finding is supported by other cord blood studies demonstrating that deficient vitamin D levels increase the risk of eczema both physician diagnosed and questionnaire-based [11], [12].

The immune-regulatory properties of vitamin D exerted through e.g. promotion of pro-inflammatory cytokines and induction of regulatory T cells include an ability to shift the Th1/Th2 balance with capacity to inhibit both Th1 and Th2-type responses leading to opposite effects on disease development, i.e. enhancing vs. reducing the risk [38]. The complex interplay between the genetic makeup, the early life milieu, and the timing of sufficient vitamin D exposure during maturation of the immature immune system may determine the direction of the immune-modulatory properties of Vitamin D. Additional programming effects of fetal vitamin D exposure apart from immune-modulation might be alterations of the airway microbiome from e.g. induction of the endogenous antimicrobial peptide cathelicidin in bronchial epithelial cells [39].

Overall, our study suggests an association between deficient fetal 25(OH)-Vitamin D exposure and development of recurrent TROLS during preschool age with a sizable effect estimate. However, despite the rigorously clinically determined diagnoses in this study and the objective assessment of cord blood 25(OH)-Vitamin D, the question of causality cannot be answered by an observational study. Thus, acknowledging that and the possibility of residual lifestyle confounding, this study prompts the performance of randomized controlled trials of maternal supplementation of vitamin D during pregnancy to shed light on the possible causative and thus modifiable effect of fetal vitamin D deficiency for development of asthma, allergy and eczema. Several randomized controlled trials are currently being conducted by our group and others (clinicalTrials.gov identification numbers: NCT00856947, NCT00920621).

### Conclusion

This study shows an interdependence of 25(OH)-Vitamin D deficiency in fetal life and occurrence of recurrent TROLS during preschool in children of the at-risk COPSAC_2000_ cohort. Further research is required to establish whether vitamin D supplementation during pregnancy can prevent development and severity of such disorders.

## Supporting Information

Data S1 **Core data of the manuscript.**
(XLS)
